# Financial crisis in the framework of non-zero temperature balance theory

**DOI:** 10.1371/journal.pone.0279089

**Published:** 2022-12-22

**Authors:** MohammadReza Zahedian, Mahsa Bagherikalhor, Andrey Trufanov, G. Reza Jafari

**Affiliations:** 1 Physics Department, Shahid Beheshti University, Tehran, Iran; 2 Institute of Information Technology and Data Science, Irkutsk National Research Technical University, Irkutsk, Russia; Korea University, KOREA, REPUBLIC OF

## Abstract

In financial crises, assets see a deep loss of value, and the financial markets experience liquidity shortages. Although they are not uncommon, they may cause by multiple contributing factors which makes them hard to study. To discover features of the financial network, the pairwise interaction of stocks has been considered in many pieces of research, but the existence of the strong correlation between stocks and their collective behavior in crisis made us address higher-order interactions. Hence, in this study, we investigate financial networks by triplet interaction in the framework of balance theory. Due to detecting the contribution of higher-order interactions in understanding the complex behavior of stocks we take the advantage of the order parameter of the higher-order interactions. Looking at real data of the financial market obtained from *S*&*P*500 index(SPX) through the lens of balance theory for the quest of network structure in different periods (on and off-crisis) faces us with the existence of a structural difference of networks corresponding to the periods. Addressing two well-known crises the Great regression (2008) and the Covid-19 recession (2020), our results show an ordered structure forms in the on-crisis period in the financial network while stocks behave independently far from a crisis. The formation of the ordered structure of stocks in crisis makes the network more resilient to disorder (thermal fluctuations). The resistance of the ordered structure against applying the disorder measure the crisis strength and determine the temperature at which the network transits. There is a critical temperature, *T*_*c*_, in the language of statistical mechanics and mean-field approach which above, the ordered structure destroys abruptly and a first-order phase transition occurs. The stronger the crisis, the higher the critical temperature.

## Introduction

Financial crises that are a period of reduced economic activity and ongoing hardships consist of a sharp drop in international trade and rising unemployment and it has happened for as long as the world has had currency. Taking advantage of theories to model the financial network to figure out how financial crises develop and how they could be prevented, faces us with a complex system which is not easily predictable. In the last century, a number of market crashes have happened all over the world, but there are some important financial crises that have impacted significantly on people’s lives, namely, the Great Depression in 1929 [1] and the Great Recession in 2008 [2]. Market crashes have been investigated with the aim of understanding [3, 4] and predicting [5]. In [6] authors have addressed the collective behavior of markets in the crisis and they benefited the Vicsek model to represent an explanation for this complexity. Due to answer to a relevant question that how government can play a vital role in economic recovery, Bahrami *et al*. [7] used the Ising model to analyze the hysteresis of the economic network and they suggested a threshold that the recovery bill should be bigger than that. In addition, one can construct a signed weighted network of the financial market using correlation matrix [8–10] and interaction matrix [9] with the help of huge economic data stored in the last decades. In the context of a financial market network, the nodes and links represent companies and their connections, respectively. The sign and weight of each link show the type and strength of the two connected companies. The constructed network can be analyzed by different models such as percolation [3] and random theory [11, 12]. These models can help us to find new features and reveal hidden patterns in data. There are many investigations in different branches of science from financial network [10, 13] to biological network [14, 15] and brain network [16] that consider only pairwise interactions in order to find the hidden features of a complex system. Despite all efforts and dedicated methods that address the crisis, it seems that it is still unforeseeable; this research aims to study financial networks in the framework of balance theory to address higher-order interaction in the quest of discovering whether considering higher-order interaction can differentiate the structure of the financial network near and far from the crisis? Or how the structure of the network can be affected by a crisis?

An outstanding theory that goes beyond pairwise interactions and considers higher-order ones is the Heider Balance theory that has been initially proposed by Fritz Heider who used it in the psychological science [17]. Then the theory extended to signed networks by Cartwright and Harary [18] and applied to other fields such as politics [19], ecology [20], and social media [21]. Balance theory is based on triadic groups of relationships in which every two individuals have an animosity (−1) or a friendship (+1) relationship. By considering three-body interactions and the two possibility of signs for each relation, four different configurations of triangles can occur as follows: the triads with three positive relationship (+ + +), two positive relationship (+ + −), one positive relationship (+ − −), and three negative relationship (− − −). Triads with an even (odd) number of negative relationships are called balanced (unbalanced). The main purpose of the balance theory is to decrease the tension and lessen the number of unbalanced triads in a network of relations and move the system toward a balanced state. The balanced state of a network occurs if and only if all signs of relations in the network are positive (heaven) or two cliques create that individuals in each clique have positive relations and all of the relations between two cliques are negative (bipolar). Dynamics of balance theory probes how a network starting from a random initial state moves to heaven or bipolar [22–24].

Authors in [22, 25] proposed the discrete time and continuous time model, respectively, to study the dynamics of the balance theory on a fully connected network. The dynamic of a network of three-body interaction in nonzero temperature has been studied in [26] that uses the balance theory in a fully connected network with weighted triangles whose weights are coming from Normal distribution. Marvel *et al*. [27] defines the energy landscape for networks to reveal that networks may be trapped in the local minima so-called, jammed states, which their structure depends on the size of the network. Belaza *et al*. investigate the balance theory from the statistical physics point of view [19] and model the triadic energy and tested it on data set of relations between countries during the Cold War era. Balance theory also managed to find application in brain networks to differentiate the brain networks of participants with autism spectrum disorder from healthy control [28]. Besides, investigations on balance theory that focus on the properties of the real world data; Estrada *et al*. [29] introduced a method to measure the degree of balance and find that social networks have a low degree of balance. Also [21] calculate the global level of balance of a very large online social network.

While there are many investigations on financial markets [9, 13, 30], understanding financial crisis using the market’s network under higher-order interaction is the main purpose of this study. In fact, we are looking for the hidden structure of a market by going beyond pairwise interactions in order to study the collective behavior of markets. To this end, we use models of statistical physics that worked in the past properly [26, 31, 32]. Actually, we believe that understanding the financial markets is beyond the simple averaging of indexes such as the overall index and the annual return. In this paper, we aim for understanding the financial markets and financial crises using the market network. To do so, we specify crisis periods (on-crisis) and normal periods (off-crisis) in the observational data and model the market network (MN) by triadic interactions of companies, forming triangle interactions, then we utilize the balance theory on the MN to investigate the behavior of the market in different periods of time, and compare results of crisis periods and normal periods. We calculated the balance theory’s statistical parameters as a function of temperature and analyzed them from the financial point of view. The results show there is a big difference between on-crisis and off-crisis periods in some characteristics such as the number of balanced triads, the energy of the network, and the critical temperature. We use the *S*&*P*500 index(SPX) and we have chosen 40 companies randomly to study the behavior of the financial market in general.

Using the time series of those companies and their correlations, we construct a financial network of the market in several time windows. After the construction of the financial network, we run the balance theory on the correlation network of stocks and we seek the dynamics of the constructed network in non-zero temperature.

## Materials and methods

### From data description to network construction

**Fig 1:**
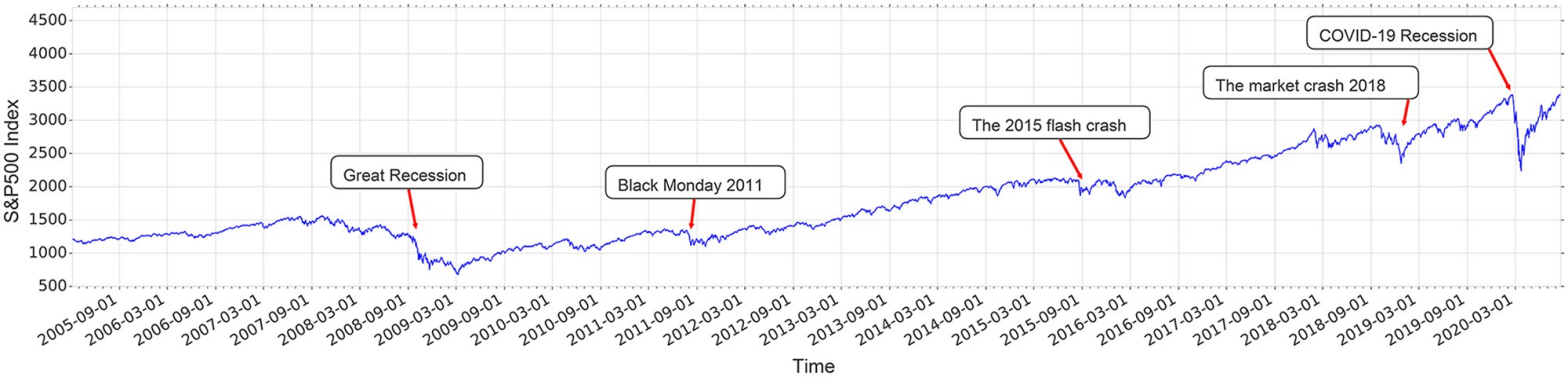
Crisis index. Time series of *S*&*P*500 index with highlighted crises.

Financial markets are made by buying and selling numerous stocks and other types of financial instruments. Therefore, the statistical behavior of stocks that describes the micro-states of a market in different periods can shed light on the macro-states that emerge in the market. Crisis as an unforeseeable financial event in which the value of assets will drop significantly demonstrates that the fluctuation in the value of stocks percolates all over the market. A financial network is a complex system in which its nodes represent the stocks, and links represent the correlation between stocks which can be used to predict the global properties of the financial market. We got the *S*&*P*500 index(SPX) and we used the closed price of the daily return of 40 companies of the index that have been chosen randomly from distinct industries to investigate generally the impact of the crisis on the financial market, in the time periods of 2005 to 2020. The regulated closing price of stocks is prepared by yahoofinance [33]. We analyze four specific time widows. October 1,2008—December 10,2008 and January 29,2020—April 8,2020 are close to the financial crisis and we call them on-crisis 2008 and 2020. Also, July 28,2005—October 6,2005 and November 15,2018—January 30,2019 are far from the financial crisis and are called off-crisis 2005 and 2019 respectively. During the periods which we have selected, there are two prominent financial crises the great recession in 2008 [7, 34], and the COVID-19 recession in 2020 [35]. Fig 1 illustrates the behavior of the *S*&*P*500 index over time and different crises have been marked in it. The two well-known crises that we want to address are the 2008 Global Financial crisis, the Great Recession, and an ongoing global economic recession that is a direct result of the COVID-19 pandemic, the COVID-19 Recession.

**Fig 2:**
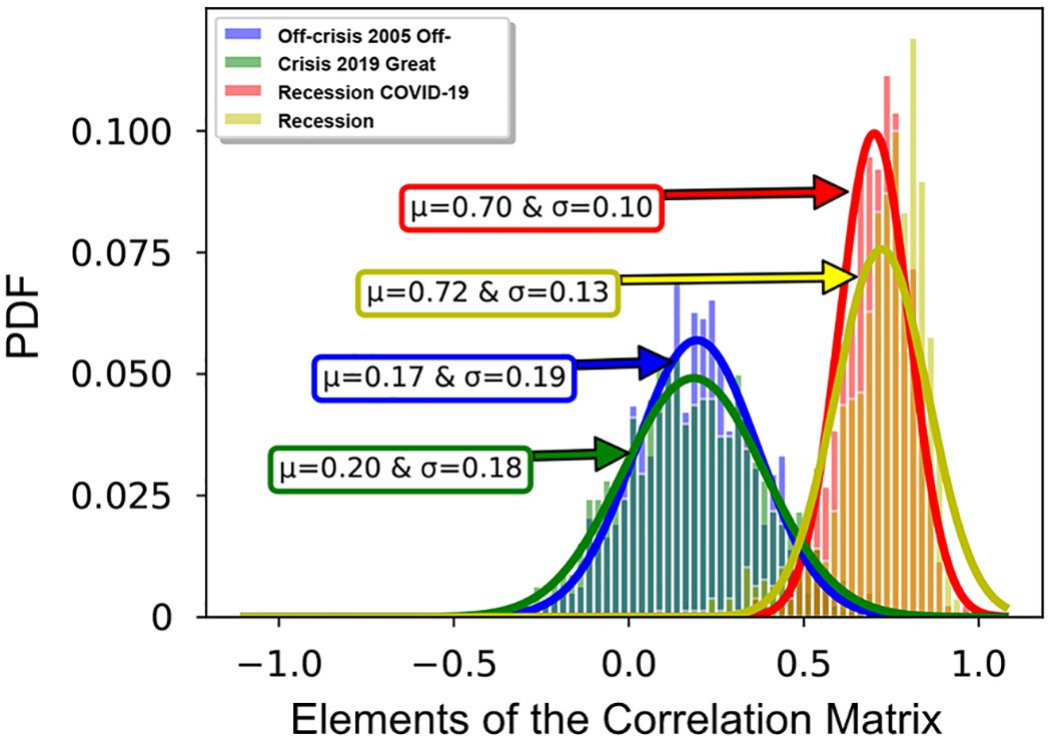
Probability distribution in on and off crisis periods. The probability distribution function (PDF) of correlation matrix for four periods of time. Fitted curves to off-crisis 2005(blue), off-crisis 2019(green) both have small mean values in regards to on-crisis periods which is a representation of weak correlation of stocks in off-crises periods. The positive mean values of curves in the on-crises periods, Great Recession 2008(red), Covid-19 Recession 2019(yellow), demonstrate the strong correlation between stocks.

We use the correlation matrix of stocks in which each element values between [−1, +1] and represents the correlation between two stocks. By considering the time series of the companies, we calculate the log-return of the daily closing price of the *i*^*th*^ company in time *t*, *x*_*i*_(*t*)

$$\begin{array}{c} x_{i}\left(t\right)=\text{log}\;P_{i}\left(t\right)-\text{log}\;P_{i}\left(t-1\right), \end{array}$$ (1)

where *P*_*i*_(*t*) is the daily closing stock price of company *i* at time *t*. Now we can extract the correlation among stocks in time period (*t*, *t* + *τ*), in which *τ* is 50 (we have 77 time-windows that each window is for a period of 50 days). For each time-window, we calculate the correlation matrix, **C**:

$$\begin{array}{c} C_{ij}=\frac{1}{\sigma_{i}\sigma_{j}}{\left\langle \left(x_{i}\left(t\right)-\mu_{i}\right)\left(x_{j}\left(t\right)-\mu_{j}\right)\right\rangle }_{win(t,t+\tau )}, \end{array}$$ (2)

where *C*_*ij*_ is an element of the correlation matrix representing the relation between two stocks *i*, *j* and 〈⋯〉_*win*(*t*, *t*+*τ*)_ is the time average over the time-window from *t* to *t* + *τ*, and *μ*_*i*_ is the average of *x*_*i*_(*t*) over the time-window defined as 〈*x*_*i*_(*t*)〉_*win*(*t*,*t*+*τ*)_, and *σ*_*i*_ is the standard deviation of *x*_*i*_(*t*) defined as $\sqrt{{\langle {\left(x_{i}\left(t\right)-\mu_{i}\right)}^{2}\rangle }_{win(t,t+\tau )}}$. Fig 2 displays the probability distribution function (PDF) of elements of the correlation matrix for four periods of on and off crisis which we mentioned above. Simply, it can be seen that the PDF of elements of the correlation matrix in off-crises and on-crises are different. The fitted Gaussian curves demonstrate that in both off-crises periods and both on-crises periods, markets behave almost similar in the language of mean values, *μ*, and the standard deviations, *σ*. The small mean value of PDF in off-crises periods represents that stocks have random behavior and there is a weak correlation between them while the mean value of PDF in on-crises periods confirms that there exists a strong correlation between stocks which results in an emergence of collective behavior of stocks in crisis.

**Fig 3:**
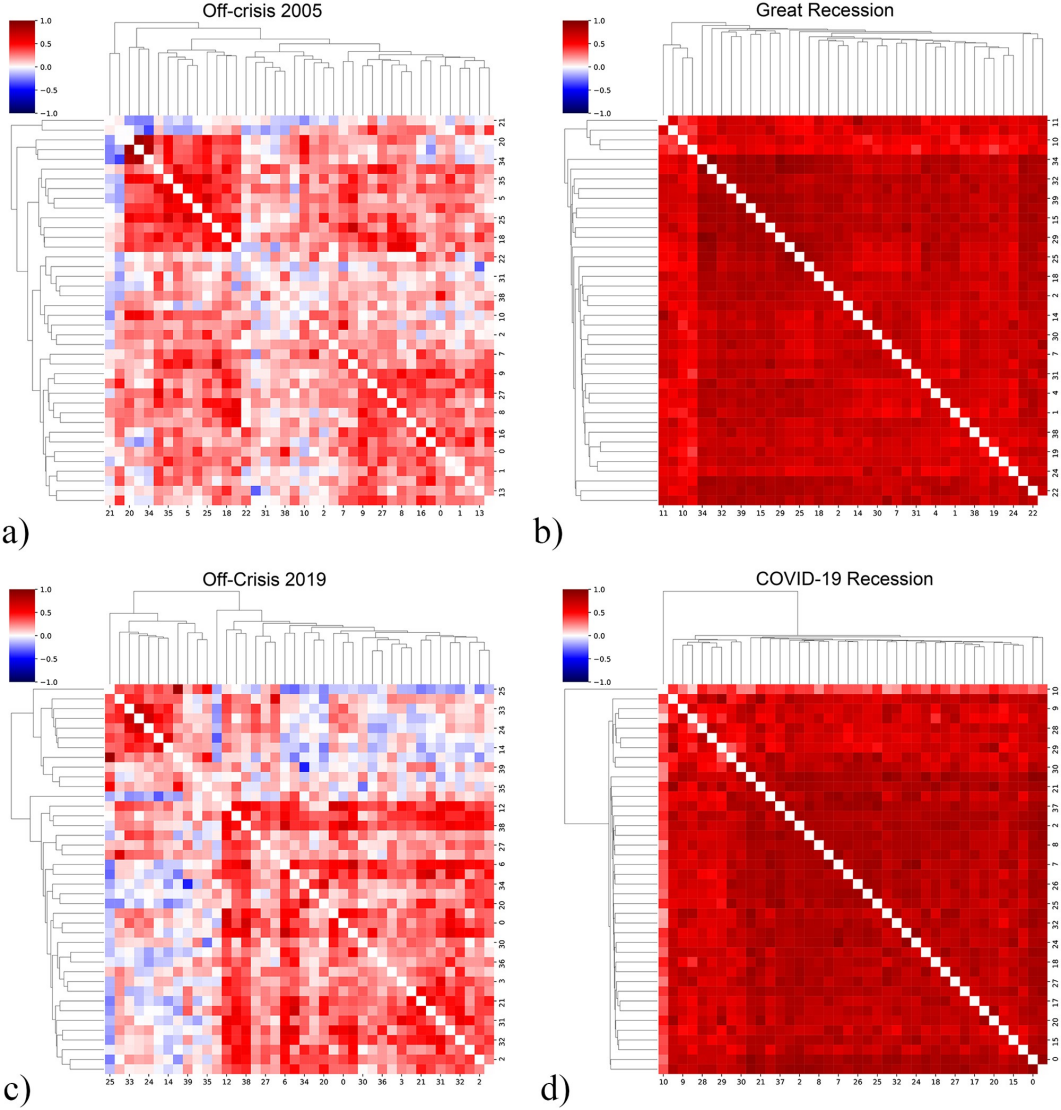
The correlation matrix of stocks in on and off crisis periods. Heatmap of the correlation matrix in a,c) Off-crisis and b,d) On-crisis periods. We use dendrogram to detect communities inside the correlation matrices and to illustrate the similarity of elements. There is no noticeable correlation between stocks in off-crisis (a,c) while in on-crisis (b,d) we see a strong correlation of the stocks.

In order to clarify how stocks are correlated in on and off crisis periods, we plot the heat-map of the correlation matrices and to provide a visual vision of the communities inside of them, we have used community detection through the dendrogram representation. Newman et al. proposed a set of algorithms for detecting community structures in a network [36]. They state that the dendrogram initially gives an overall picture of the connected network and then splits it into smaller communities. Fig 3 upper row indicates the cluster-map of correlation matrices in periods off-crisis 2005 and on-crisis 2008, Great Recession, and the lower row is the cluster-map of correlation matrices in periods off-crisis 2019 and on-crisis 2019, COVID-19 Recession. In off-crisis cases, Fig 3a and 3c stocks are behaving independently far from the crisis so correlation values are random but in on-crises, Fig 3b and 3d shows that stocks are correlated and behave collectively. This means that fluctuations in the value of stocks in a crisis are intercorrelated and occur simultaneously/sequentially.

Calculating the correlation matrix of stocks enables us to construct the financial network in which nodes represent stocks and each link states the correlation value between two nodes. In the quest of studying the impact of higher-order interactions on the dynamics of the financial markets, we construct the network based on triadic relationships. Hence, we propose a Hamiltonian of triangles whose links are elements of the correlation matrix and have continuous values. Despite of the Hamiltonian proposed for weighted balance theory which addresses a network of triangles [26] in which each link can be either +1 or −1 and triangles are weighted, here links are weighted and have values between −1 and +1. Thus, the Hamiltonian is:

$$\begin{array}{c} H=-\sum_{i>j>k}C_{ij}C_{jk}C_{ki}, \end{array}$$ (3)

in which *C*_*ij*_ is an element of correlation matrix that indicates the correlation between two stocks *i* and *j*. According to Eq (3), the energy of the network is proportional to the summation of the multiplication of the triad’s links. If the multiplication of links of a triangle is positive it is in a balanced state and increasing the number of balanced triangles results in decreasing the energy of the network and the opposite is true for unbalanced triangles. In the on-crisis period, the stocks are highly correlated and an ordered structure emerges in the network which informs the formation of a big and strong community of correlated stocks. Imposing a disorder, interpreted as temperature, can measure the resilience of this ordered structure against changes. At low temperatures, the network insists to keep its structural order unless there is a critical temperature, *T*_*c*_, that for temperatures above, a failure occurs in the structure of the network. So long as the correlation between stocks are strong, the disorder (thermal fluctuation) can not overcome the ordered structure so, a higher temperature is needed to get the system out of the financial crisis period and destroy the ordered structure. In the off-crisis period, markets are independent therefore the structure of the network does not show the existence of collective behavior. In this case, the critical temperature is approximately zero due to a lack of a strong correlation between stocks. Investigating the dynamics of a financial network under various temperatures is the main focus of the following section.

### Triadic interactions under balance theory in non-zero temperature

We proposed a Hamiltonian for the constructed network that considers triadic interactions. Since we know the Hamiltonian of the system, we can calculate the statistical quantities by Boltzmann-Gibbs distribution *p* ∼ *e*^−*βH*(*x*)^, where *H*(*x*) is the Hamiltonian. Boltzmann-Gibbs distribution helps us to derive all possible moments of the system to investigate their statistical behavior in equilibrium. Statistical mechanics in addition to mean-field approximation is our desired approach to understand the dynamics of the system statistically. In general, three quantities can be calculated, the mean value of the link, the two-star (two links with a common node), and the energy which is the mean value of the Hamiltonian. Using equilibrium statistical mechanics and mean-field methods, we need to derive the partition function. For a canonical ensemble that is classical and discrete, the canonical partition function is defined as $Z=\sum_{i}e^{-\beta E_{i}}$ where *i* is the index for microstates of the system, *β* is the thermodynamic quantity proportional to the inverse of the temperature *1*/*T*, and *E*_*i*_ is the total energy of the system in the respective microstate. The system under study is constructed based on the correlation matrix in which its elements are weighted and reflect the dependency of stocks on each other; encourages us to use the weighted balance theory model that has been introduced in [26] comprehensively. Here by applying the following changes we can correspond the Hamiltonian 3 with one being studied in [26]. The multiplication of the absolute value of correlation matrix elements represents the weight of triangles, *J*_*ijk*_ = |*C*_*ij*_| * |*C*_*jk*_| * |*C*_*ki*_|, and the sign of each component represent a link of the network, *S*_*ij*_ = sgn(*C*_*ij*_). Via the applied changes the Hamiltonian Eq 3 can be rewritten in the following form:

$$\begin{array}{c} H=-\sum_{i>j>k}J_{ijk}S_{ij}S_{jk}S_{ki}, \end{array}$$ (4)

where *J*_*ijk*_ is the weight of a triangle built on nodes *i*, *j*, *k* and *S*_*ij*_ is the link between nodes *i*, *j* that can be either −1 or +1. Using the mean-field approach enables us to calculate the statistical quantities of the system.

#### Mean value of link

According to mean-field method the Hamiltonian Eq 4 can be divided into two parts, *H* = *H*_*ij*_ + *H*′, where *H*_*ij*_ includes all terms in the Hamiltonian that contain *S*_*ij*_, and *H*′ is the rest of the Hamiltonian that relates to other links, {*S*′}, [26].

$$\begin{array}{cc} & H_{ij}=-S_{ij}\sum_{k\neq i,j}J_{ijk}S_{jk}S_{ki},\\ & p\equiv \left\langle S_{ij}\right\rangle =\sum_{S_{ij}=\left\{\pm 1\right\}}S_{ij}\;P\left(S_{ij}\right)=\left(1\right)\times P\left(S_{ij}=1\right)+\left(-1\right)\times P\left(S_{ij}=-1\right)\\ & =\frac{1}{Z}\sum_{\left\{S^{\prime }\right\}}e^{-\beta H^{\prime }}\sum_{S_{ij}=\{\pm 1\}}S_{ij}e^{-\beta H_{ij}}\\ & p=\text{tanh}(\beta \left\langle \sum_{k\neq i,j}J_{ijk}\;S_{jk}\;S_{ki}\right\rangle ), \end{array}$$ (5)

where *Z* = ∑exp(−*βH*) is the partition function and the term in the parentheses is called the mean value of weighted two-stars, *Q* ≡ 〈∑_*k*≠*i*,*j*_ *J*_*ijk*_*S*_*jk*_*S*_*ki*_〉, and is interpreted as the mean-field each link feels.

#### Mean value of two-star

Following the same process mentioned above the Hamiltonian can be divided in the form of, *H* = *H*_*jk*,*ki*_ + *H*″, where *H*_*jk*,*ki*_ consists of all term including *S*_*jk*_ and *S*_*ki*_, and *H*″ is the rest, [26].

$$\begin{array}{cc} & H_{jk,ki}=-S_{jk}\left(\sum_{l\neq i,j,k}J_{jlk}S_{jl}S_{kl}\right)-S_{ki}\left(\sum_{l\neq i,j,k}J_{kil}S_{kl}S_{il}\right)-J_{ijk}S_{ij}S_{jk}S_{ki},\\ & q\equiv \left\langle S_{jk}S_{ki}\right\rangle =\sum_{S_{jk},S_{ki}=\left\{\pm 1\right\}}S_{jk}S_{ki}\;P\left(S_{jk}S_{ki}\right)=\frac{e^{2\beta Q}-2\;e^{-2\beta J_{ijk}\text{tanh}\left(\beta Q\right)}+e^{-2\beta Q}}{e^{2\beta Q}+2\;e^{-2\beta J_{ijk}\text{tanh}\left(\beta Q\right)}+e^{-2\beta Q}}\\ & =q(Q,J_{ijk},\beta ), \end{array}$$ (6)

the fraction represents that *q* is the function of the weighted mean value of two-star, *Q*, temperature, and the weights, *J*_*ijk*_ that have been assumed are coming from Gaussian probability distribution. The detailed process of preceding analytical solutions has been completely done in [26]. Considering continuous values of weights, we change the summation in the definition of *Q* to integral, $\sum_{k\neq i,j}\to \int_{-\infty }^{\infty }dJ\;P\left(J\right)$, and we integrate over all weights that are coming from Gaussian probability distribution to calculate the mean field each link feels due to weighted two-stars over it.

$$\begin{array}{c} Q\equiv \left\langle \sum_{k\neq i,j}J_{ijk}S_{jk}S_{ki}\right\rangle =\left(N-2\right)\int_{-\infty }^{\infty }J^{\prime }\;P\left(J^{\prime }\right)\;q\left(Q,J^{\prime },\beta \right)\;dJ^{\prime }=f\left(Q,\mu ,\sigma ,N,\beta \right). \end{array}$$ (7)

The result is a function of the mean value and the variance of the Gaussian probability distribution. The coefficient, *N* − 2, is the normalization parameter and It is the number of two-stars on a specific link. While here we represent an analytical solution by the assumption of Gaussian probability distribution of weights, in working with real data the probability distribution of weights is inferred from data. Solution of the self-consistence Eq 7, which is called an order parameter(*Q*), confirms that there is a critical temperature, *T*_*c*_, determines the state of the system. For the temperatures above, *T* > *T*_*c*_, the network is in a random state of positive and negative links whereas for the temperature below the critical, *T* < *T*_*c*_, the network is in a balanced state whether paradise (all links are positive) or bipolar (a division of the network into two groups that are friendly relationship inside and enmity between groups). Therefore, the system experiences a phase transition as the result of a sudden jump between the ordered structure and the random state. In other words, the resistance of the ordered structure under applying thermal fluctuations can be measured by an order parameter. Since stocks behave similarly in the crisis periods, an ordered structure forms that is resistance against disorder and higher temperature is needed for the network to make a transition. Therefore the stronger the crisis, the higher the critical temperature. Investigating the behavior of statistical quantities of the constructed network based on real financial data in different periods of time is going to be discussed and illustrated in the next section.

### Critical temperature on and off crisis

Introducing a Hamiltonian that describes the dynamics of a system and considering a parameter, temperature (*T*), that can be interpreted as tension and randomness allows a system to fluctuate and provides us the chance of studying a system by Boltzmann-Gibbs statistics. Theoretical solution and derivation of the self-consistence Eq 7 tell us there is a critical temperature, *T*_*c*_, that network is in an unbalanced state for temperature higher than *T*_*c*_ and in a balanced state for temperature lower than *T*_*c*_ and the system experiences a first order phase transition. To confirm this result by simulation, we use the correlation matrices that are constructed directly from real data as the initial state of the network; Then we run a Metropolis Monte Carlo simulation on the initial network to study its evolution at different temperatures. According to the dynamics, we choose a link by chance and switch the sign in order to increase the number of balanced triads; This is equivalent to reducing the energy of the network. If switching the sign of a link lessens the energy of the network, we will accept this change, and the sign of the link is updated, otherwise, we will accept this change by a Boltzmann probability proportional to *exp*(−*β*Δ*E*) where Δ*E* is the energy difference, (*E*_*f*_ − *E*_*i*_), after and before flipping the sign of the randomly selected link. For both initial networks whether on-crisis or off-crisis periods, for *T* > *T*_*c*_ the structure is random, but the magnitude of the *T*_*c*_ depends on the initial conditions. The behavior of the order parameter, *Q*, versus temperature is a suitable quantity to illustrate the differences in the dynamics of the financial network in on-crisis and off-crisis periods and their structure.

**Fig 4:**
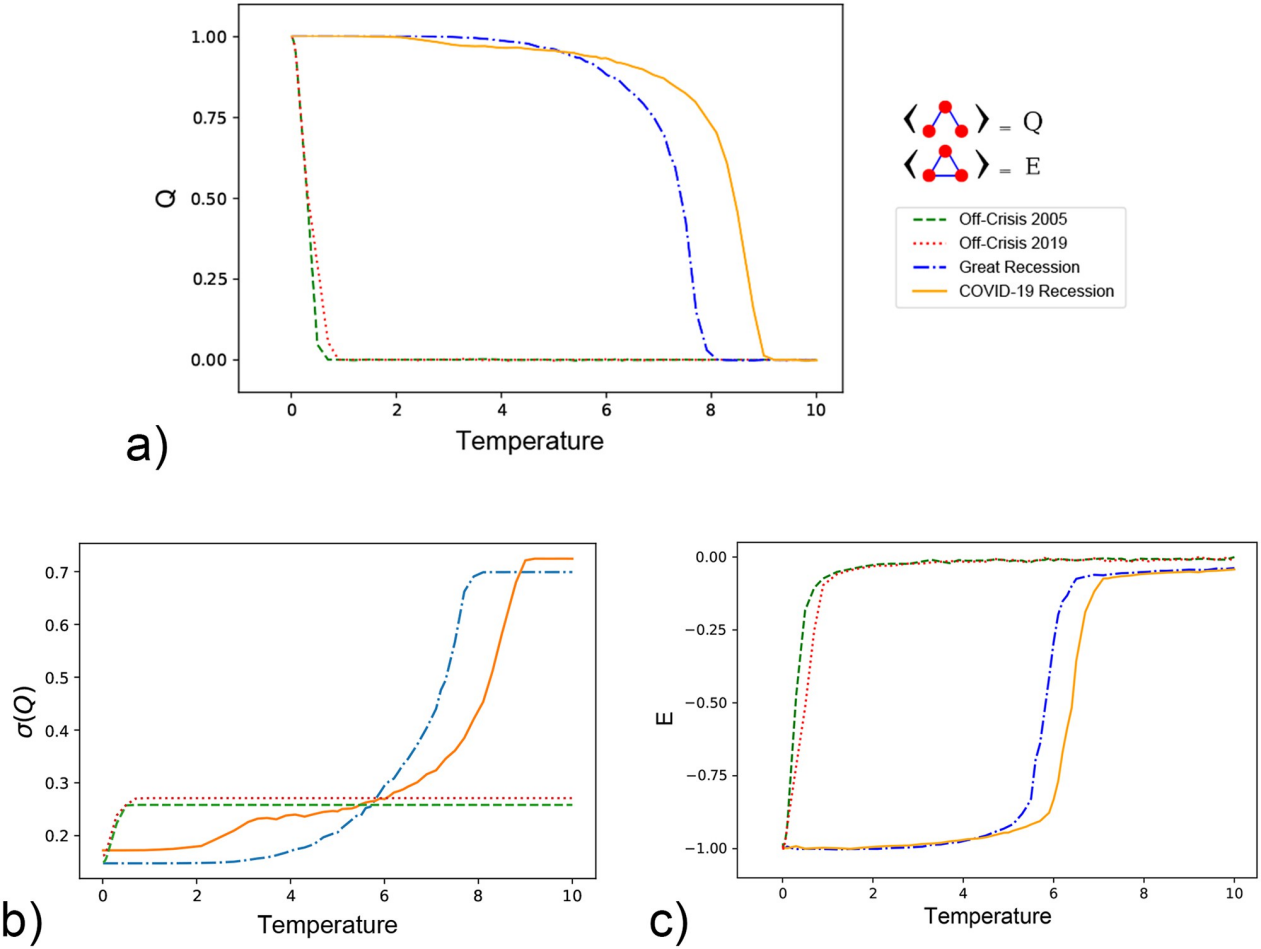
The behavior of order parameter, Q, and *σ*(*Q*), and the Energy versus temperature. a) The mean value of weighted two-stars, *Q*, versus temperature in two different on-crisis periods (the left figure). The *Q* equals 1 in low temperature while increasing temperature, *T*, will result in destroying the ordered structure of the network and zero value of the *Q* in the random configuration of the network. In off-crisis periods the value of *Q* is different from zero around approximately zero temperatures and it will quickly reduces to zero due to the weak correlation between stocks, b) The standard deviation(std) of the two-star weights versus the temperature (the middle figure). The std is larger for *T* > *T*_*c*_ in both on and off-crisis periods than *T* < *T*_*c*_, and in on-crisis with respect to off-crisis we see bigger values of std; this is caused by the difference in correlations, c) The energy of the network versus temperature (the right figure). In the on-crisis, the structural order of the network resists against changes hence it is needed to increase the temperature to a critical value that can overcome the ordered phase. In off-crisis periods the structure of the network is not in an ordered phase so a small amount of disorder can push the network in a random configuration and zero value of energy.

Fig 4a demonstrates the order parameter, *Q* (mean value of weighted two-star), in on-crisis and off-crisis periods; the presence of the strong correlation between stocks in the on-crisis indicates the existence of collective behavior among stocks and the formation of an ordered structure in the network. In the ordered phase *Q* = 1 which states that all triangles in the network are in the balanced state (heaven). Applying disorder, increasing temperature, can not change the ordered structure until the point where the ordered phase of the network disrupts in a critical temperature, *T*_*c*_, and its consequence is *Q* = 0. In off-crisis periods, due to the lack of strong correlation between stocks, the critical temperature is approximately zero and increasing temperature results in a random structure of the network and zero value of *Q*. The collective behavior of the network in the on-crisis period prevents the system from going to a random phase so higher thermal fluctuation is needed to overcome the ordered structure of the network, this results in a bigger critical temperature with respect to the off-crisis period. Fig 4b depicts the standard deviation(std) of the two-star weights versus the temperature. The plot shows that in the temperatures higher than the critical temperature we have a larger std with respect to the temperatures lower than the critical temperature whether in the crisis or non-crisis periods. Also, for temperatures higher than the critical temperature, the std for the crisis periods has bigger values rather than non-crisis periods. Fig 4c illustrates the energy of the network versus temperature in two periods of on-crisis and off-crisis. In the ordered phase which all triangles of the network are in the balanced state the total normalized energy of the system equals −1 according to Eq 3. Although the ordered phase preserves its structure even by applying disorder, there is a critical point, *T*_*c*_, in which above the structure destroys abruptly. Increasing temperature leads the structure of the network toward a random configuration of balanced and unbalanced triangles and zero value of the energy of the network. The difference in critical temperature of two on-crises periods states that the COVID-19 Recession is a stronger crisis with respect to the Great Recession in 2008 since it needs more disorder to get the system out of financial crisis due to the strong correlation of stocks.

**Fig 5:**
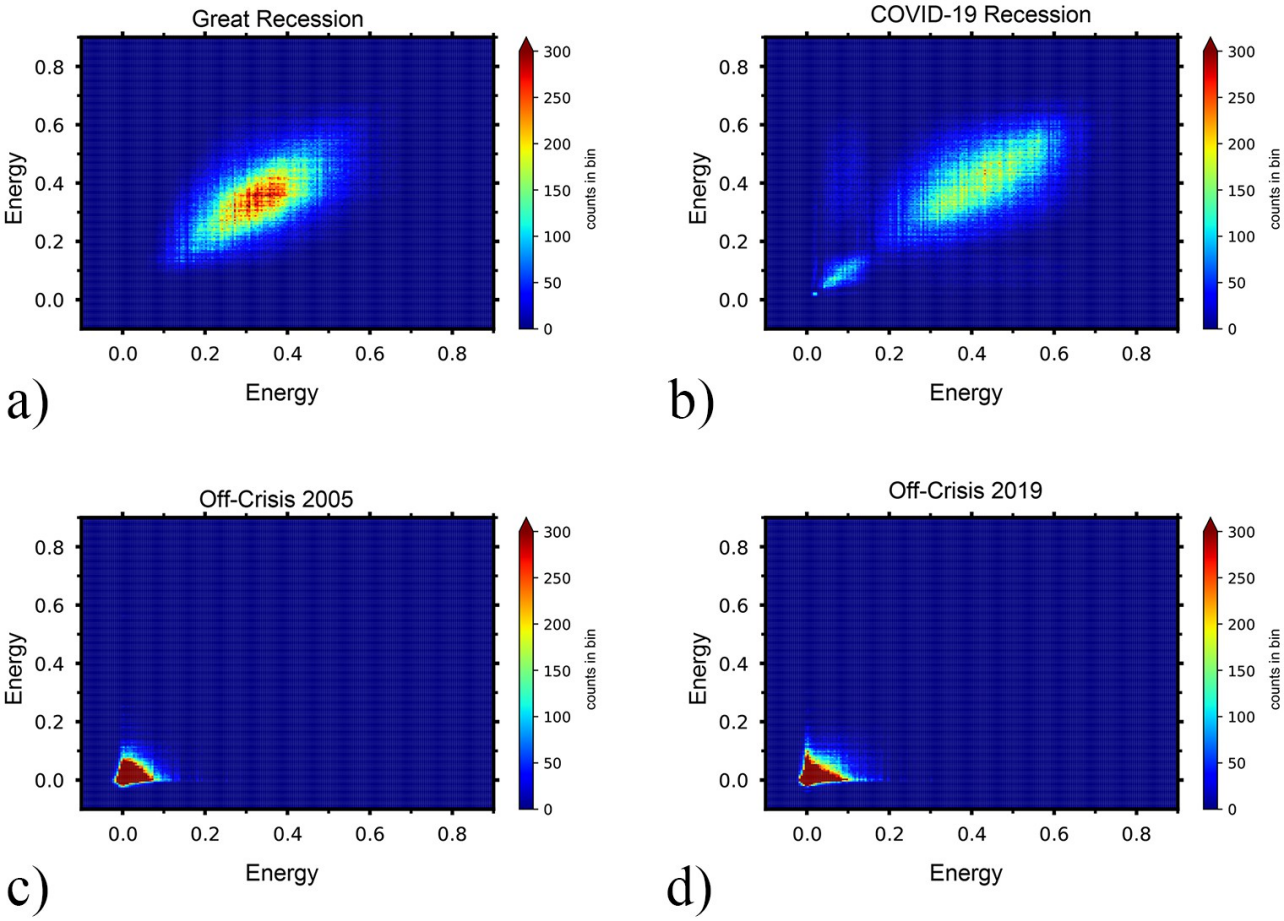
The pattern of energy-energy landscape between triads with one shared link. The upper row shows the energy-energy landscape for a) the Great Recession and b) the COVID-19 recession. The lower row is the energy-energy landscape for two off-crises periods c) July 28, 2005- October 6, 2005, and d) November 15, 2018 –January 30, 2019. Each point is representative of the number of triangles with a common link which have the specified values of energies. In the on-crises periods, triangles with various energies are connected while in off-crises periods points are localized to small energies.

In the off-crisis periods, the critical temperatures are near zero which means stocks are weakly correlated and behave independently so the energy of the network equals zero. Fig 5 shows the energy landscape of triangles that have a joint link. Each triangle, *u*, has a specific energy, *E*_*u*_ and if two triangles *u* and *v* share a common link, we would consider the (*E*_*u*_, *E*_*v*_) as a point in the energy-energy plot. To clarify the method, we use 2D-histogram to count the number of triangles that exists in the different area of the energy-energy landscape. It is easy to see that in the on-crisis 2008(Great Recession) and on-crisis 2020(COVID-19 Recession), triangles with a common link have more energy and a wide pattern of connections. However, the pattern of connections of triangles is localized in the off-crisis periods. Note that, because of the localization of energy in the off-crisis, there are bins in which the abundance of triangles that have joint links is much more (80000 connected triangles) than in the on-crisis (300 connected triangles). So, for better illustration, we use an extended 2D histogram. Actually, bins with an abundance of more than 300 are colored red.

**Fig 6:**
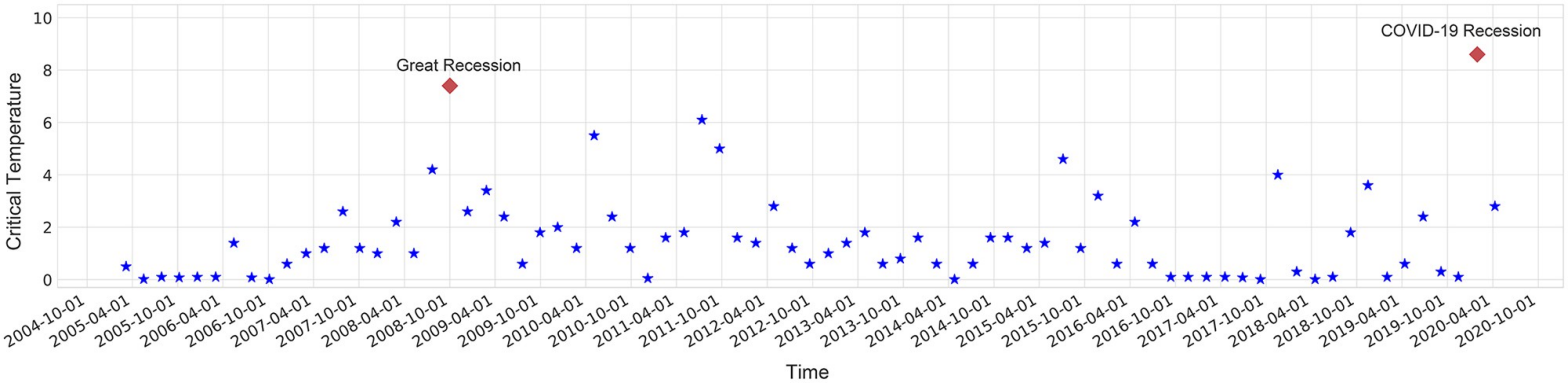
Critical temperature for Great Recession and COVID-19 Recession. The stronger correlation of stocks results in a more serious crisis and a higher thermal fluctuation needed to destroy this highly correlated structure of stocks.

Fig 6 shows the critical temperature for several time windows. We have divided the whole time period from 2005–2020 into windows, each one is for a period of 50 days and the critical temperature has been calculated in each window. Surrounding the crisis points the presence of correlation leads to an increase in the critical temperature. Actually, the collective behavior of stocks in crisis results in an ordered structure that is resistant to disorder hence higher temperature is needed to get the system out of this situation. In the figure, we highlight the outstanding crises with a red diamond. One can see that the critical temperature in crisis periods is more than the times that are far from a crisis.

## Discussion and conclusion

**Fig 7:**
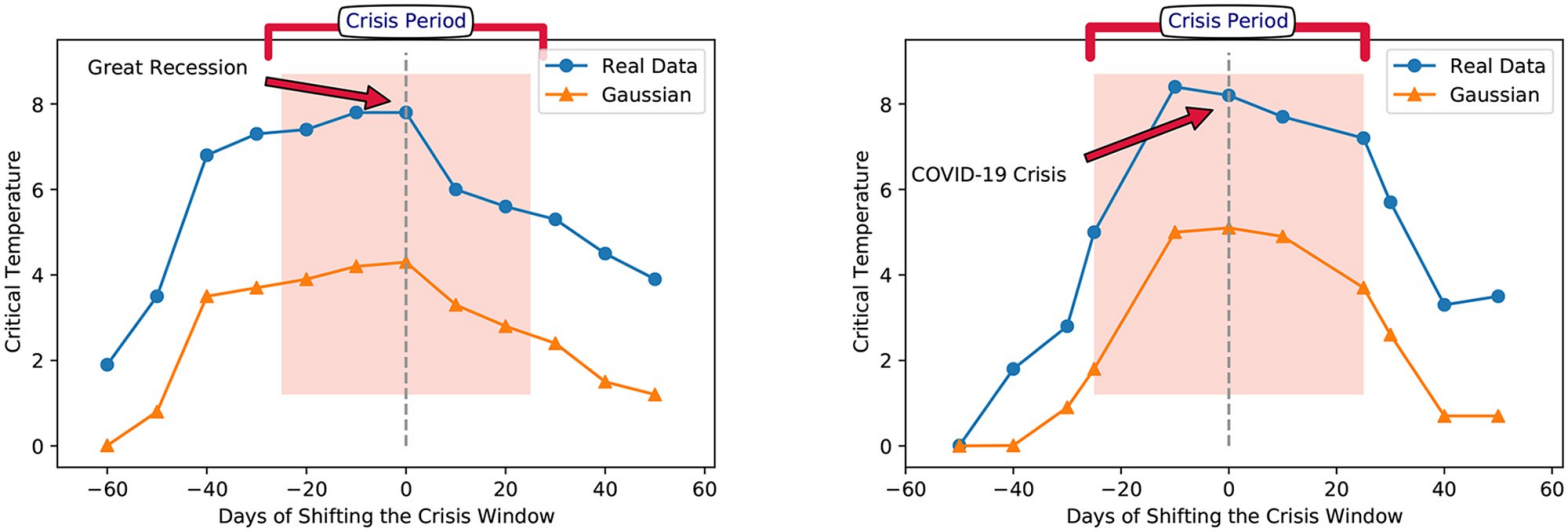
The efficiency of order parameter, Q, versus the correlation in figures a), and b) we consider a moving window that moves from 50 days below the critical point (crisis) to 50 days above and we calculate the critical temperature in each interval of the moving window. The result of studying the structure of the network by the moving window shows that the proposed order parameter is more accurate in predicting crises. It predicts the happening of the crisis sooner and with more intensity rather than correlation.

The similar behavior of stocks during the crisis period and the complex behavior of financial networks in crisis has been devoted our attention to the essential role of higher-order interactions. In a crisis correlations of stocks are not only dependent but also behave collectively. To better understand this collective behavior, higher-order interactions will be helpful. Here, inspired by the weighted balance theory, we introduce an that addresses the correlation of correlations and confirms that the system experience a phase transition. The functionality of the order parameter, Q, versus temperature, T, displays that in on-crisis periods the network is more resistant to disorder than in off-crisis periods due to the structure made by correlation of correlations in the network. Even comparing the critical temperate of two crises tells us that the ordered structure formed during the COVID-19 Recession is more resilient to disorder with respect to the Great Recession since it has a higher critical temperature (*T*_*c*_(*COVID* − 19*Recession*) > *T*_*c*_(*GreatRecession*)). To make clear the usefulness of our order parameter in detecting the crisis, we simulated a correlation network with the random Gaussian weights with the same mean/variance as real data. The results show that our order parameter, which is based on considering higher-order correlations, is a more sensitive quantity in predicting the occurrence of a crisis than correlation. Fig 7 apparently displays that while the simulated random Gaussian correlation senses the crisis is happening, our order parameter, the mean value of weighted two-stars, is more sensitive than Gaussian in the case of time and value. It predicts that the crisis will happen sooner and with more intensity. The higher-order correlation of stocks demonstrates the structure of the financial network in crisis periods is more resilient than in off-crisis periods against applying a disorder (thermal fluctuations). This evidence brings us to conclude that higher-order interactions have a key contribution to the consideration of the structure of the crisis.
